# Genetic Variant rs7758229 in 6q26–q27 Is Not Associated with Colorectal Cancer Risk in a Chinese Population

**DOI:** 10.1371/journal.pone.0059256

**Published:** 2013-03-12

**Authors:** Lingjun Zhu, Mulong Du, Dongying Gu, Lan Ma, Haiyan Chu, Na Tong, Jinfei Chen, Zhengdong Zhang, Meilin Wang

**Affiliations:** 1 Department of Oncology, The First Affiliated Hospital of Nanjing Medical University, Nanjing, China; 2 Department of Genetic Toxicology, The Key Laboratory of Modern Toxicology of Ministry of Education, School of Public Health, Nanjing Medical University, Nanjing, China; 3 Department of Environmental Genomics, Jiangsu Key Laboratory of Cancer Biomarkers, Prevention and Treatment, Cancer Center, Nanjing Medical University, Nanjing, China; 4 Department of Oncology, Nanjing First Hospital, Nanjing Medical University, Nanjing, China; University of Pennsylvania, United States of America

## Abstract

**Background:**

A recent genome-wide association study has identified a new genetic variant rs7758229 in *SLC22A3* for colorectal cancer susceptibility in a Japanese population, but it is unknown whether this newly identified variant is associated with colorectal cancer in other populations, including the Chinese population.

**Methods:**

We examined the associations between rs7758229 and colorectal cancer risk among 1,147 cases and 1,203 controls matched by age and sex. Logistic regression model was used to assess the associations.

**Results:**

No significant association was found between rs7758229 and colorectal cancer risk (OR = 0.95, 95%CI  = 0.84–1.09, *P* = 0.463). Similar results were observed in the stratification of tumor location (OR  = 0.94, 95%CI = 0.80–1.11, *P* = 0.481 for colon cancer, and OR  = 0.96, 95%CI  = 0.82–1.13, *P* = 0.621 for rectum cancer).

**Conclusions:**

Our findings did not support an association between rs7758229 in 6q26-q27 and the risk of colorectal cancer in a Chinese population.

## Introduction

Colorectal cancer is the leading cause of cancer-related death in most countries, which is caused by a combination of genetic and environmental risk factors [1], [2], [3]. Twin studies have shown that genetic susceptibility accounts for ∼35% of disease etiology, most of which is still unclear [4]. Recent widespread availability of high-throughput genomic approaches have provided the opportunity to scan the genomes of large numbers of individuals in genome-wide association studies (GWAS) rapidly [5]. Several GWAS have been completed with the aim of identifying genetic variants influencing the risk of colorectal cancer [6]. Previously, three validation [7], [8], [9] studies in Chinese failed to replicate most of GWAS-identified loci, suggesting that genetic heterogeneity existed between Caucasians and Asians.

Recently, Cui et al. conducted a GWAS in a Japanese population (4,809 colorectal cancer cases and 2,973 controls) and identified a new locus rs7758229 in *SLC22A3* for distal colon cancer [10]. Moreover, they found cumulative effects of rs7758229, other genetic and environmental factors could increase colorectal cancer risk. However, little or nothing is known about whether the effect of this locus exists in other Asian populations. Here, we carried out an independent case-control study to assess the association between rs7758229 identified by Cui et al. and colorectal cancer risk in a Chinese population.

## Materials and Methods

### Study population

The patients of colorectal cancer were recruited from September 2010 at the First Affiliated Hospital and Nanjing First Hospital of Nanjing Medical University, which have been described in detail previously [11]. All the cases were histologically confirmed colorectal adenocarcinoma. The pathological stage of colorectal cancer was classified into Dukes A, B, C, and D. Tumor grade was divided into low, intermediate, and high. The control subjects were randomly selected from a pool of more than 25,000 cancer-free individuals on the basis of physical examinations and frequency-matched to cases on age and sex. The exclusion criteria included no history of cancer. After having signed informed consent, each subject donated 5 ml of blood for genomic DNA extraction. This study was approved by the institutional review boards of Nanjing Medical University.

### Genotyping

Genomic DNA was isolated from peripheral blood lymphocytes. In this study, rs7758229 was genotyped using the TaqMan assay (Applied Biosystems). The sequences of primer and probe for each SNP are available on request. Genomic DNA of 50 ng and 0.5×mix (TaKaRa Bio, JPN) was used for each reaction and amplification was performed under the following conditions: 50°C for 2 min, 95°C for 10 min followed by 45 cycles of 95°C for 15 sec, and 60°C for 1 min. We assessed genotype data quality by typing 10% blinded replicate samples; the concordance rate was 100.0%.

### Statistical analyses

Hardy-Weinberg equilibrium of the controls' genotype distributions was tested by a goodness-of-fit chi-square test. Unconditional univariate and multivariate logistic regression analyses were performed to obtain crude and adjusted odds ratios (ORs) for risk of colorectal cancer and their 95% confidence intervals (CIs). A *P* value <0.05 was considered statistically significant, and all statistical tests were two sided. Statistical analyses were done using SAS software, release 9.1 (SAS Institute, Cary, NC).

## Results

A total of 1,147 colorectal cancer cases and 1,203 control subjects were included in this study (Table 1). The mean age was 60.1 years old for cases and 59.9 years old for controls. Cases were more likely to have family members with cancer than controls (21.2% versus 10.6%, *P*<0.001). The observed rs7758229 genotype frequencies among the control subjects were in agreement with the Hardy-Weinberg equilibrium (*P* = 0.778). As shown in Table 2, the frequencies of GG, GT and TT genotypes were 58.3%, 35.6%, and 6.1%, respectively, among the cases, and 56.4%, 37.6%, and 6.0%, respectively, among the controls. Furthermore, rs7758229 T allele frequency was 0.248 among the cases and 0.238 among the controls, and the difference was not statistically significant (OR = 0.95, 95%CI = 0.84–1.09, *P* = 0.463). We performed a stratification analysis according to tumor location (colon and rectum) to examine the association between rs7758229 and colorectal cancer risk. Similarly, rs7758229 T allele was not associated with the risk of colon cancer or rectum cancer, compared with the G allele (OR = 0.94, 95%CI = 0.80–1.11, *P* = 0.481 for colon cancer, and OR = 0.96, 95%CI  = 0.82–1.13, *P* = 0.621 for rectum cancer).

**Table 1:** Characteristics of colorectal cancer cases and controls.

Variables	Cases (*n* = 1147)		Controls (*n* = 1203)	
	N	%	N	%
Age (mean ± SD)	60.1±12.6		59.9±14.3	
Sex				
Male	702	61.2	698	58.0
Female	445	38.8	505	42.0
Family history of cancer				
No	904	78.8	1076	89.4
Yes	243	21.2	127	10.6
Tumor site				
Colon	559	48.7		
Rectum	588	51.3		
Dukes stage				
A	97	8.4		
B	494	43.1		
C	422	36.8		
D	134	11.7		
Tumor grade				
Low	85	7.4		
Intermediate	880	76.7		
High	182	15.9		

**Table 2:** Genotype and allelic frequencies of rs7758229 among cases and controls and associations with risk of colorectal cancer.

Chr. 6q26-q27	Controls	Total colorectal cancer cases			Colon			Rectum		
	N (%)	N (%)	OR (95% CI)^a^	*P* a	N (%)	OR (95% CI)^a^	*P* a	N (%)	OR (95% CI)^a^	*P* a
rs7758229										
GG	679 (56.4)	669 (58.3)	1.00		325 (58.1)	1.00		344 (58.5)	1.00	
GT	452 (37.6)	408 (35.6)	0.92 (0.77-1.09)	0.308	203 (36.3)	0.94 (0.76-1.16)	0.547	205 (34.9)	0.89 (0.72-1.10)	0.292
TT	72 (6.0)	70 (6.1)	0.98 (0.69-1.39)	0.915	31 (5.6)	0.90 (0.58-1.40)	0.631	39 (6.6)	1.06 (0.70-1.60)	0.777
T allele^b^	0.248	0.238	0.95 (0.84-1.09)	0.463	0.237	0.94 (0.80-1.11)	0.481	0.241	0.96 (0.82-1.13)	0.621

a Adjusted for age and sex in logistic regression model.

b Additive model.

## Discussion

In the present study, we found no statistically significant association between rs7758229 in *SLC22A3* and colorectal cancer risk, and no association with the risk of tumor location.

Our inability of replicate the findings of Cui et al. [10] may be due to several reasons. First, a weak and inadequate power may explain our inconsistent results. However, this study had reasonable power (>75%) to detect an OR of 1.28 per copy of T allele, which had been reported previously by Cui et al. [10]. Second, we recruited colorectal cancer cases from hospitals and selected controls from populations, which might not well represent the whole population and might result in potential selection bias. Third, it is biologically conceivable that the same susceptibility variant for colorectal cancer may be implicated across different populations. Therefore, the discrepant findings may be related to genetic and/or environmental modifiers that vary in frequency between the Chinese populations and the Japanese populations. Finally, Cui et al, reported that rs7758229 had a more significant association with the risk of distal colon cancer (*P* = 7.92×10^−9^) than colorectal cancer (*P* = 1.31×10^−5^) [10], suggesting that their carcinomas have different pathological mechanisms of carcinogenesis involving different genetic and epigenetic defects [12].

In conclusion, our study confirmed that rs7758229 in 6q26-q27 may not contribute to the risk of colorectal cancer in a Chinese population. Therefore, further other loci and large-scale GWAS of colorectal cancer in the Chinese population are expecting.
